# Oncogenic Human Papillomavirus involvement in epithelial ovarian carcinoma among women in Morocco

**DOI:** 10.6026/97320630015055

**Published:** 2019-02-03

**Authors:** Rahma Ait Hammou, Mustapha Benhessou, Amal Bouziyane, Najwa Hassou, Mohammed Nabil Benhchekroun, Hlima Bessi, Moulay Mustapha Ennaji

**Affiliations:** 1 Laboratory of Virology,Microbiology, Quality and Biotechnologies/Eco-toxicology and biodiversity,faculty of Sciences and Techniques, Mohammedia,University Hassan II of Casablanca,BP:146 Mohammedia 20650, Morocco; 2 Universite Mohamed VI des Sciences de la sante of Casablanca, Morocco; 3 Faculty of Medicine and Pharmacy of Casablanca,University Hassan II of Casablanca, Morocco

**Keywords:** Oncogenic Human Papillomavirus, fresh biopsies, epithelial ovarian carcinoma, viral carcinogenesis, Genotyping, PCR typespecific

## Abstract

Epithelial Ovarian cancer (EOC) although rare is the most lethal gynecological cancer in women worldwide. Despite its high prevalence few studies have been performed to evaluate the prevalence and determinants of HPV infection worldwide. The aim of the present study was to investigate the presence of HPV-DNA in Moroccan patients with EOC using PCR among women in Casablanca, and to examine the prevalence of some HPV genotypes in Moroccan population. We performed a study of HPV detection on Fresh biopsies of 70 epithelial ovarian cancer patients. PCR was realized using the MY09/11 and GP5+/6+ primers. Genotyping of HPV was performed by PCR typespecific for HPV 6, 11, 16, 18, 31, and 33.Data was statistically analyzed using SPSS software. Hence, the mean age was 48.9 years (range,21-76 years). Serous adeno carcinoma (75.71%) and stage III of the disease represent the majority of cases. eight patients were HPV positive (11.42%).Results of HPV genotyping revealed predominance of two genotypes: HPV 16 (87.5%) and HPV 31(12.5).No co-infection identified. Approximately 75% of positive cases had a serous cystadeno carcinoma and more than 62,5% had FIGO advanced stage (III or IV).Our study showed that high-risk HPV infection could play a major role among patients with EOC in Morocco.

## Background

EOC although rare is the most lethal gynecological cancer in women worldwide and represent the fifth most common malignancy, leading to 22.400 newly diagnosed cancer cases and over 14,300 deaths every year in the US Good management of EOC depends on an early detection of the disease; unfortunately, the late and poor prognosis is a result of (a) the nature of this malignancy which is insidious asymptomatic in its early onset, (b) deficiency of effective methods of detection at an early stage of the disease, and (c) resistance of chemotherapy [1].

In Morocco, ovarian cancer is the 5th female cancer and accounts for 4, 9 % of cases recorded during the period 2008-2012, in terms of incidence this malignancy represent brute incidence of 5.6 per 100,000 women, standardized incidence on the Moroccan population of 5.4 per 100,000 women and a standardized incidence on the world population of 6.2 per 100,000 women [2] with more than 70% of cases discovered in advanced stages of the disease (III or IV). More than half of the cases occur between 45 and 64 years with serous adeno carcinoma as the predominant histological type [3]. Efforts for early detection of epithelial ovarian tumors are ineffective, because of the ambiguity of both the origin and the pathogenesis of this malignancy.

Infectious agents, mainly viruses, are among the few known causes of cancer and contribute to a variety of malignancies. Human papilloma virus (HPV), Hepatitis B and C virus, and Epstein bar virus, are implicated in the cervical cancer, the liver cancer and Burkitt's lymphoma respectively as found by Moss et Blaser [4]. The Link between HPV and the genesis of cervical cancer has been indisputably proven and identified [5], whereas, its involvement in ovarian cancer still controversial despite the anatomical proximity of the two organs. Some studies provide evidence of the involvement of the viral agents in the carcinogenesis [6,7] and others deny it [8,9]. Thus, the role of HPV in ovarian cancer tumorigenesis remained a big challenge. Because the ovarian cancer etiology is multi-factorial and HPV infection is related to sexual behavior, a more precise assay should take the covariates such as age, ethnicity, and lifestyle into consideration. The aim of the present study was to determine viral etiology of EOC especially HPV, thus we investigate the presence of HPV DNA in Moroccan patients with EOC using the highly sensitive technique of polymerase chain reaction (PCR) among women in Casablanca area, Morocco and we examine the prevalence of some HPV genotypes in Moroccan population.

## Methodology

The study involved a series of 70 fresh biopsies from patients aging from 21 to 76 years, with histo pathologically confirmed EOC obtained after surgical intervention in the department of gynecology and obstetrics "A", Ibn Rochd University Hospital, Casablanca, Morocco. Informed consent was obtained from all participants and the study protocol was approved by the local ethics committee of Faculty of Medicine and Pharmacy of Casablanca Morocco.

The fresh biopsies harvested by a clinician, were immediately placed into 2 mL cryo tubes and stored in liquid nitrogen. Histological type of tumor was determined according to the criteria of the World Health Organization (WHO) [10] and the stages were established according to the International Federation of gynecology and Obstetrics (FIGO) criteria [11]. The molecular analysis using PCR was performed at LVMQ/ETB of FSTM.

DNA Extraction was performed using the phenol/chloroform method routinely used in the LVMQB/ETB. Briefly, small sections of 5µm obtained from frozen tissue samples using a scalpel, were placed in 1.5 mL sterile Eppendorf tube. Depending on the size of the biopsy, 250 to 500µL of lysis buffer (10mM Tris-HCl pH 7.5, 10mM EDTA, 10%SDS) containing 200µg/ml of proteinase K were added and digested for 3 h at 55°C. Purification was performed by phenol/chloroform/iso amyl alcohol (25:24:1). Then the DNA was precipitated using 7.4 M ammonium acetate and absolute ethanol. The DNA pellet was washed in 70% cold ethanol and dried at 37°C for 15 min before being re-suspended in 30 to 50µL of ultrapure water and stored at-20°C until PCR amplification.

Before the PCR amplification of the viral DNA, the extract was dosed in Nano drop 8000 Spectrophotometer (Nano drop Technologies, Wilmington, DE, USA).To evaluate the efficiency of the extraction, integrity of specimen and absence of PCR inhibitors, all extracted DNA were subject to an amplification of β-globin reference gene using the primers pair PCO4/GH20 as described previously [12]. All amplification was carried out with 100 ng/µl of DNA in Perkin Elmer 2400 GeneAmp PCR thermal Cycler (Scientific Support, Inc, Hayward, CA). DNA from the SiHa cell line was used as positive PCR control and ultrapure water as negative control.

**Figure 1:**
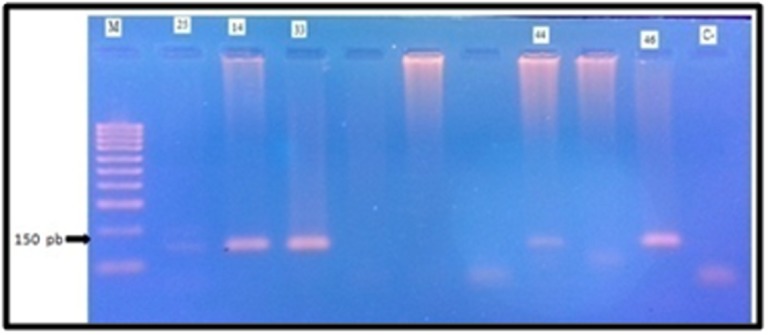
Representative illustration of HPV Detection Electrophoresis gel photo (A). Lanes 14,25 ,33;44;46 correspond to HPV positive specimens; C-: negative control (sterile distilled water); C+: Positive control ); M: 100 bp ladder molecular weight marker.

HPV detection and typing was performed using a nested PCR using the MY09/11 and GP5+/6+ consensus primers (Invitrogen Life Technologies, Frederick, MD, USA). These primers amplify respectively 450 bp and 150 bp sequences of the L1 gene of HPV encoding for the major capsid protein [13]. MY09/11 PCR was performed using 24µL of Mix [1X PCR buffer (Promega), 800µM dNTP, 250µM MgCl2, 0.4µM MY09/11 primers and 0.05U/µl of Taq polymerase (Promega)] supplemented with 1µL of DNA for a final volume of 25µL.The nested PCR GP5+/6+ were performed with 2µL MY09/11 PCR product using the same composition of Mix, expect the use of: 20pmol of GP5+/6+ primers, 100µM of MgCl2 and 400µM of dNTP. Temperature profile used was: initial denaturation at 94°C for 10 min, 35 cycles of 94°C/1min for denaturing, 55°C (MY09/11) / 40°C (GP5+/6+)/1 min and 72°C/1 min for elongation, 72°C/7 min for final incubation. All PCR products were analyzed using migration on agarose gel 2% stained with Ethidium bromide and visualized by UV light (Serva, and Heidelberg, Germany). A representative gel is given in Figure 1. Typing was carried out type-specific PCR for HPV 6, 11, 16, 18, 31 and 33 as described previously [13]. Statistical analysis for obtained data was performed using SPSS software. P-Values are calculated using tree-ways ANNOVA. The significance level was considered when p < 0.05.

## Results

70 patients were included in this study. The mean age at the time of admission was 48.9 years (range, 21-76 years). The histopathological diagnosis revealed 53 patients with serous cyst adenocarcinoma (75.71%), nine (12.85%) had mucinous type, five (7.14%) had endo-metrioid and tree (4.30%) had an undifferentiated adenocarcinoma. The distribution of patients according to FIGO stage was as follows: 4 (5.71%) stage-I, 16 (22.85%) stage-II, 47 (67.14%) stage-III and 3 (4.30%) with stage IV as given in Table 1.

**Figure 2:**
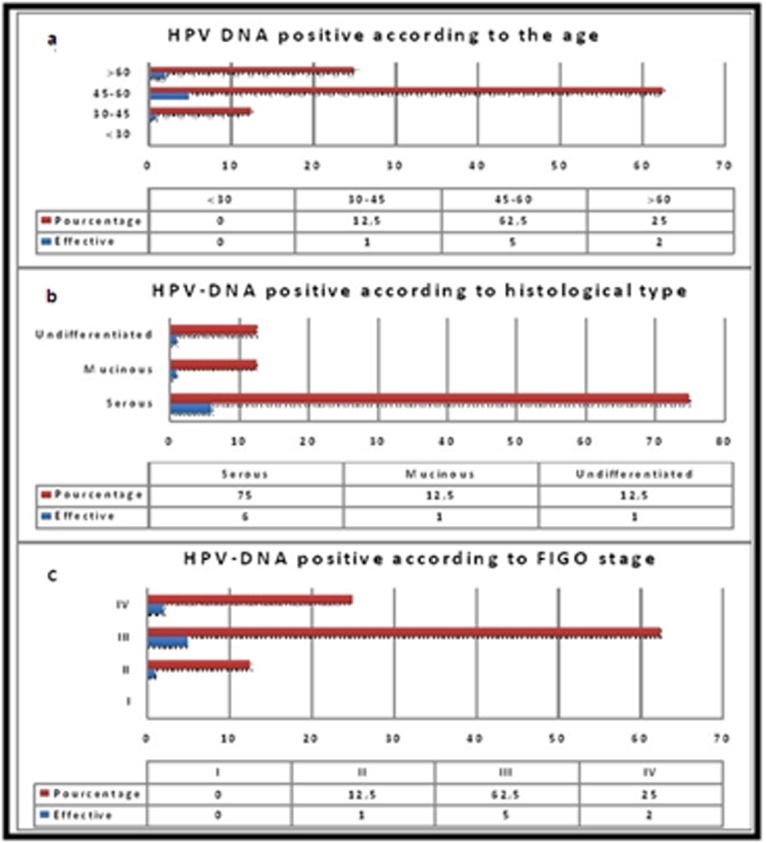
Distribution of HPV-DNA positive cases according to age and clinical features.a: HPV DNA Positive according to the age. b: HPV DNA positive according to histologic type.c: HPV DNA positive according to FIGO stage. Abbreviations: FIGO: International Federation of Gynecology and Obstetrics.

**Figure 3:**
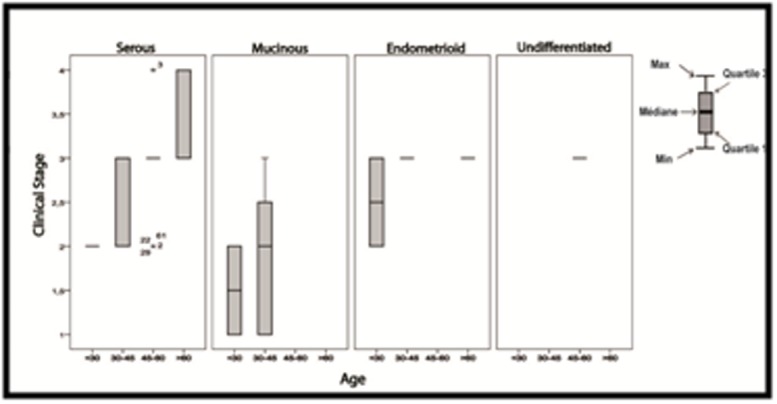
Relative expression of the validated HPV which have significantly different expression in EOC tumor samples. Boxes represent the sample distribution with the mean, vertical lines mark the 10th percentile, and outliers are represented as dots. P-values are calculated via Tree-Ways ANOVA ,using the SPSS software (Statistical Package for the Social Sciences).

Between 70 EOC samples analyzed, HPV-DNA was detected in 11.42% (8/70) of cases. Only two high-risk genotypes were identified: HPV 16 was the most prevalent with 87.5% (7/8) followed by HPV 31 with 12.5% (1/8). None of the patients had more than one type of HPV (Table 2). The distribution of positive cases according to the age, histological type and FIGO stage is reported in Figure 2. Moreover, these results shows that more than 80% of HPV positive patients were 45 years and older exclusively for HPV type 16. 75% of positive cases had a serous cyst adenocarcinoma and more than 62.5% of positive cases had FIGO advanced stage (III or IV) of the disease. The only patient with HPV Type 31 had mucinous cyst adeno carcinoma. There was no statistical association between clinical features and HPV infection (p>0.05).Whereas, statistical analysis showed that there is correlation between age and clinical stage (p < 0.05) (Figure 3).

## Discussion

The first study on the association between HPV and ovarian cancer was published [15] and it was retracted a year later. Controversially, some studies have confirmed the relationship between HPV and the ovarian cancer [6,16,14,18], others have rejected this hypothesis [16,17,19,20]. In this current cohort, we investigated the presence of HPV DNA in 70 frozen epithelial ovarian carcinoma tissue from women in the region of Casablanca, Morocco. We used a highly sensitive method of detection, i.e. polymerase chain reaction. Several studies have also used this molecular method of investigation [16,6,21]. In contrast to some previous studies [7,8] additional information was obtained in order to establish a possible association between positivity of HPV-DNA and various clinical parameters. Despite the adoption of high sensitivity method, the viral HPV DNA was detected in only 11.42% of cases of epithelial ovarian carcinoma. These results are similar to those found by several authors [16,6]. We identified HPV types 16 in 87.5% (7/8) of total positive cases, followed by HPV types 31 in 12.5% (1/8) of cases. Thus, HPV 16 was the leading subtype found in this study. Its predominance is reported in almost all studies of this type worldwide [16,6,22]. This present study also detected the presence of HPV types 31, whereas HPV 33 is most associated with HPV 16 in other studies. Although HPV type 18 or 33 are also the most recognized beside HPV 16 in the ovarian cancer case [22,23] in our cohort we identified a single HPV type 31. This type phylogenetically very close to HPV 16 is often associated with the latter in HPV virus-induced cancer cases. Moreover, this study showed that the majority of positive cases (75%) are serous cyst adeno-carcinoma as demonstrated by several other authors previously. Atalay or Wu, showed 10.5% and 62% of HPV positivity in patients with serous cyst adeno-carcinoma respectively [24,16].

In addition, in view of the investigation we cannot establish a causal relationship between HPV positivity and this malignancy. The presence of HPV-DNA in different EOC does not seem to be correlated to the histological type of tumor, the stage of development or the patient's age (p> 0.05). However, several hypotheses have been advanced to explain the presence of HPV DNA and certain oncogenic high-risk types HPV in the EOC. Most frequently cited are among others: (i) The low intensity of the DNA bands on the electrophoretic analysis of the PCR product may reflect a latent infection, which is characterized by a few copies of HPV genome in the nuclei of infected cells and therefore cannot be considered responsible of disease [25]; (ii) The geographic and host genetics may play a role in susceptibility to HPV infection [24]. (iii) Endo-metrium and fallopian tubes are a continuation of anatomical endo-cervical glands, and the infection can spread this way or (iv) the sperm may be responsible for this transition by absorbing HPV DNA and transmit these nuclear entities to cells of the reproductive system or may be carriers of the virus during passage of the endocervical canal and thus reach the ovarian cortex after ovulation [26]. In sum, although the relationship between HPV and malignant tumors of the upper genital tract remains controversial, the involvement of viruses in carcinogenesis even in the most unsuspected cancers is becoming increasingly evident.

## Conclusion

The present study showed for the first time, the presence of highrisk HPV in Moroccan patients with an epithelial ovarian carcinoma, suggesting that this virus may play an important role in ovarian carcinogenesis considered as a tumorigenic virus. These studies will be interesting to develop advanced tools for early diagnosis and a better prognosis of cancer. In view of the findings, it may be interesting to examine the possible link HPV-EOC in the large case-control studies among Moroccan patients to better elucidate this association.

## Figures and Tables

**Table 1:** Patients tumor characteristics (n=70)

Characteristics	effective	Percentage
Age: mean age= 48,9 +/- 11,9		
<30	6	8.57
30-45	17	24.29
45-60	39	55.71
>60	8	11.43
Total	70	100
Histologic Type (WHO)		
Serous cystadenocarcinoma	53	75.71
Mucinous cystadenocarcinoma	9	12.86
Endometrioid carcinoma	5	7.14
Undifferentiated carcinoma	3	4.29
Clinical stage (FIGO)		
I	4	5.71
II	16	22.86
III	47	67.14
IV	3	4.29

**Table 2:** Characteristics and HPV-type distribution of positive patients (n=8)

Patients	Age	Histology	FIGO stage	HPV-Type
1	42	Mucinous	III	31
2	48	serous	II	16
3	58	serous	IV	16
4	52	Undifferentiated	III	16
5	76	serous	III	16
6	69	serous	IV	16
7	54	serous	III	16
8	59	serous	III	16
